# Case Report: Development of Type 1 Autoimmune Pancreatitis in an Adolescent With Ulcerative Colitis Mimicking Pancreatic Cancer

**DOI:** 10.3389/fped.2021.791840

**Published:** 2021-11-26

**Authors:** Sujin Choi, Hae Jeong Lee, An Na Seo, Han Ik Bae, Hyung Jun Kwon, Chang Min Cho, So Mi Lee, Byung-Ho Choe, Ben Kang

**Affiliations:** ^1^Department of Pediatrics, School of Medicine, Kyungpook National University, Daegu, South Korea; ^2^Department of Pediatrics, Samsung Changwon Hospital, Sungkyunkwan University School of Medicine, Changwon, South Korea; ^3^Department of Pathology, School of Medicine, Kyungpook National University, Daegu, South Korea; ^4^Department of Surgery, School of Medicine, Kyungpook National University, Daegu, South Korea; ^5^Department of Internal Medicine, School of Medicine, Kyungpook National University, Daegu, South Korea; ^6^Department of Radiology, School of Medicine, Kyungpook National University, Daegu, South Korea

**Keywords:** autoimmune pancreatitis, ulcerative colitis, inflammatory bowel disease, pancreatic cancer, IgG4

## Abstract

**Introduction:** Autoimmune pancreatitis (AIP) is a rare extraintestinal manifestation of inflammatory bowel disease (IBD) which is typically responsive to corticosteroid treatment.

**Case Presentation:** We report a case of a 17-year-old male diagnosed with ulcerative colitis who subsequently developed acute pancreatitis. Blood tests demonstrated elevated pancreatic enzyme levels of amylase (1319 U/L) and lipase (809 U/L). Abdominal computed tomography revealed peripancreatic fat stranding and the presence of a perisplenic pseudocyst. Azathioprine and mesalazine were stopped as possible causes of drug-induced pancreatitis. However, pancreatic enzymes remained elevated and corticosteroid treatment was started. Despite corticosteroid therapy, amylase and lipase levels continued to increase. Infliximab was started due to a flare in gastrointestinal symptoms of ulcerative colitis. Follow-up abdominal ultrasonography revealed a pancreatic tail mass. Tumor markers, including CA 19-9, were elevated and atypical cells were seen on histological examination of an endoscopic ultrasonography-guided fine needle aspiration biopsy. Surgical pancreaticosplenectomy was performed for suspected pancreatic neoplasm. Surprisingly, histology revealed chronic pancreatitis with storiform fibrosis and infiltration of IgG4-positive cells, compatible with AIP type 1. Thereafter, pancreatic enzymes gradually decreased to normal levels and the patient has been in remission for 9 months on infliximab monotherapy.

**Conclusion:** Pediatric gastroenterologists should keep in mind that AIP may develop during the natural course of pediatric IBD. Moreover, the development of pancreatic fibrosis may be non-responsive to corticosteroid treatment and mimic pancreatic neoplasia.

## Introduction

Pancreatitis may occur during the course of inflammatory bowel disease (IBD) (1). Factors contributing to the pathogenesis of acute pancreatitis as a complication of IBD include idiopathic disease, drugs, gallstones, duodenal inflammation, endoscopic procedures, primary sclerosing cholangitis, and autoimmune pancreatitis (AIP) (2). The development of AIP during the disease course of IBD is rare and its etiology has yet to be elucidated (3).

AIP is a rare disease that is commonly associated with adult-onset inflammatory diseases. There are two clinicopathologic types (4). Type 1 AIP is considered component of systemic immunoglobulin (Ig) G4-associated disease. It typically presents with obstructive jaundice and is more common in the elderly, males, and patients of Asian descent. Type 1 AIP is more likely to relapse than type 2 AIP. Meanwhile, type 2 AIP is known to be associated with IBD, particularly ulcerative colitis (UC). Serum IgG4 levels are typically normal and patients predominantly present with abdominal pain. Type 2 AIP is more common in young Caucasians (2, 5).

AIP type 1 is seen less frequently in IBD (6). Currently, there is scarce literature reporting the development of type 1 AIP in patients with IBD. To our knowledge, there are no previous reports of type 1 AIP in pediatric populations. Herein, we provide the first report of a case of type 1 AIP in an adolescent with UC.

We present the following article in accordance with the CARE reporting checklist.

## Case Presentation

A 17-year-old male with UC was referred to Kyungpook National University Children's Hospital due to acute pancreatitis. He had been diagnosed with UC a year prior to admission and remission had been induced and maintained by medications including prednisolone, mesalazine, and azathioprine. All drugs were sequentially stopped in consideration of drug-induced pancreatitis; however, pancreatitis recurred with development of symptoms of mild abdominal pain and intermittent hematochezia. Family and past medical histories were unremarkable.

**Figure 1:**
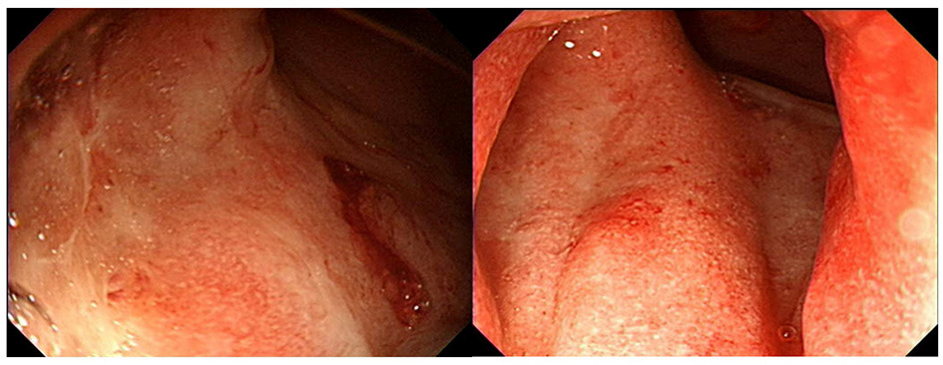
Sigmoidoscopy demonstrating superficial ulcers and erosion confined to the rectum.

**Figure 2:**
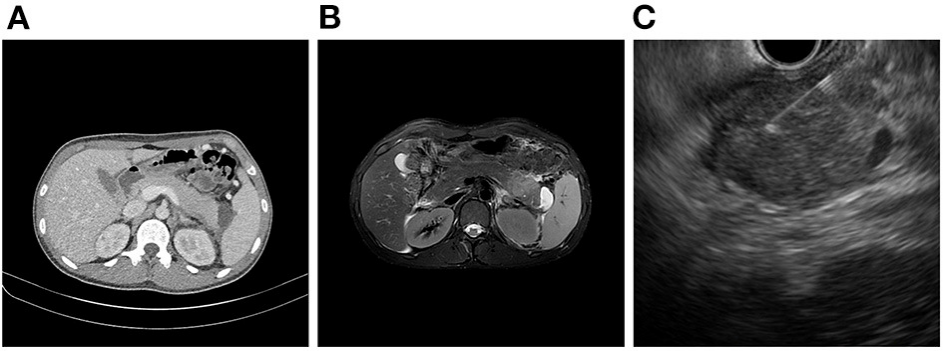
Images of **(A)** abdominal CT, **(B)** magnetic resonance imaging and **(C)** EUS-guided FNA.

On admission, his vital signs were stable and within normal limits. Physical examination revealed rapid weight loss of 23 kg over 1 year. Initial laboratory tests demonstrated the following: white blood cell count 5,170/μL; hemoglobin level, 14.7 g/dL; platelet count, 251,000/μL; erythrocyte sedimentation rate (ESR), 58 mm/h; C-reactive protein (CRP) level, 0.35 mg/dL; amylase, 1,319 U/L; and lipase, 809 U/L. Fecal immunochemical testing (FIT) was positive. The fecal calprotectin (FC) level was 814 mg/kg. Other laboratory findings, including IgG, antinuclear antibody, anti-smooth muscle antibody, and anti-mitochondrial antibody, were all within the normal range. Sigmoidoscopy showed superficial ulcers and erosions confined to the rectum (Figure 1). These findings were improved when compared to his initial colonoscopy at time of diagnosis which had revealed pancolitis. Abdominal computed tomography (CT) demonstrated peripancreatic fat stranding and a perisplenic pseudocyst (Figure 2A). Diagnostic exome sequencing of genetic mutations of *CFTR, SPINK1*, and *PRSS1*, revealed no pathological variants. No congenital abnormalities were seen on magnetic resonance cholangiopantcreatography. Methylprednisolone was started at a dose of 30 mg/day. Despite corticosteroid treatment, pancreatitis persisted and hematochezia worsened. Corticosteroids were stopped and treatment with infliximab (IFX) was started.

**Figure 3:**
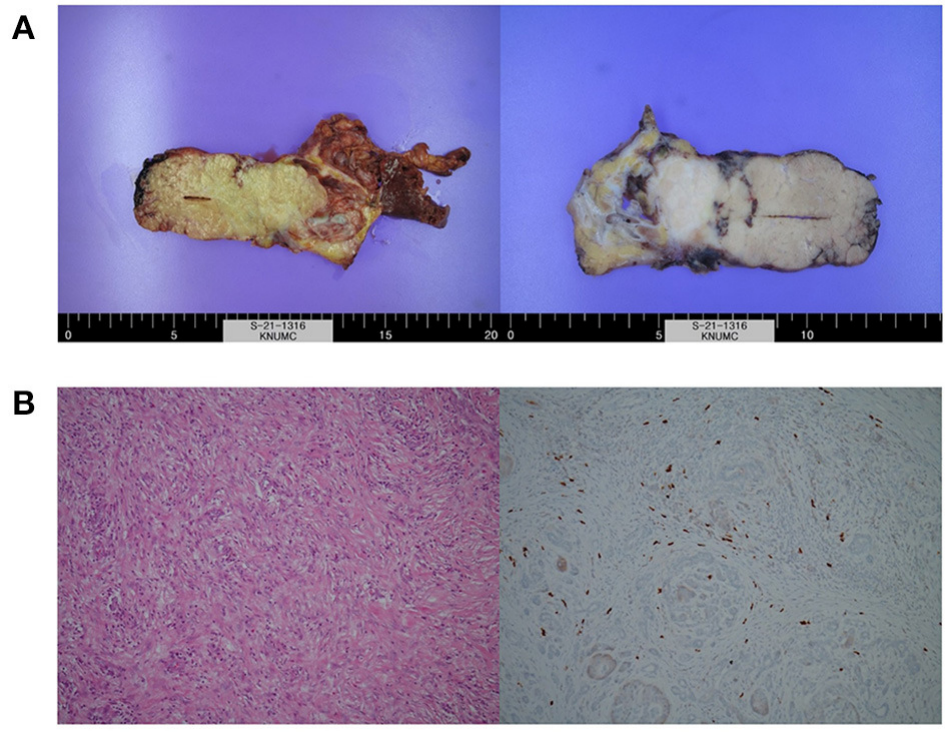
Images of **(A)** resected surgical specimens and **(B)** histologic examination showing chronic pancreatitis with storiform fibrosis and infiltration of IgG4-positive cells.

After the second dose of IFX infusion, mild decreases in amylase and lipase levels were seen. However, at the visit for his third IFX infusion, laboratory tests were as follows: ESR, 59 mm/h; CRP level, 0.05 mg/dL; amylase, 1,083 U/L; and lipase, 2,040 U/L. FIT was negative. The FC level was 63 mg/kg. Magnetic resonance imaging showed a focal mass-forming inflammatory lesion in the pancreatic tail (Figure 2B). Endoscopic ultrasound (EUS)-guided fine needle aspiration (FNA) was used to sample the pancreatic mass (Figure 2C). Histology revealed atypical cells. In addition, serum carbohydrate antigen (CA) 19-9 was elevated at 415.94 U/mL. Considering the possibility of pancreatic cancer, surgery was scheduled. Preoperative positron emission tomography-CT showed no abnormal uptake other than by the inflammatory pancreatic lesions. Chest CT was normal. Laparoscopic distal pancreatosplenectomy was conducted (Figure 3A). Histological analysis of the surgical specimen revealed chronic pancreatitis with storiform fibrosis and infiltration of IgG4-positive cells (Figure 3B).

After surgery, amylase and lipase levels decreased to 55 U/L and 45 U/L, respectively. Serum CA 19-9 normalized to 29.64 U/mL. The patient is currently 18 years old and is in clinical and biochemical remission on IFX at a dose of 5 mg/g at 8-week intervals for maintenance treatment of UC. No recurrence of pancreatitis has occurred at 9 months postoperatively.

This case report was approved by the Institutional Review Board of Kyungpook National University Chilgok Hospital (No. 2021-07-012). All procedures performed in studies involving human participants were in accordance with the ethical standards of the institutional and national research committees and with the Helsinki Declaration (as revised in 2013). Written informed consent was obtained from the patient.

## Discussion

We report a rare case of type 1 AIP in an adolescent with UC presenting with acute pancreatitis and a pancreatic mass mimicking pancreatic cancer. Surgical resection of the mass was required for both treatment and to rule out the differential diagnosis of pancreatic cancer, which revealed findings compatible with type 1 AIP. To our knowledge, this is the first reported case of type 1 AIP associated with UC in the pediatric population.

Type 2 AIP has a higher incidence in Europe and North America than in Asia (7). Meanwhile, Type 1 AIP has a higher incidence in men, while type 2 has a similar incidence between men and women (7). According to a previous study, the mean age of occurrence is 60.5 years in type 1 and 7.4 years in type 2 AIP (8) Acute pancreatitis was seen in ~30% of cases with type 2 AIP, while only 10% of type 1 AIP had acute pancreatitis. Meanwhile, 90% of type 1 AIP and 60% of type 2 AIP cases had obstructive jaundice. Unlike type 1 AIP, serum IgG4 levels are typically within the normal range in type 2 AIP, and IgG4 infiltration in organs such as the salivary glands, kidneys, and retroperitoneal membranes is not seen. Up to 30% of cases of type 2 AIP, while <4% of type 1 AIP cases, are associated with UC (8, 9). Considering the age of the patient, presentation with pancreatitis instead of obstructive jaundice, normal serum IgG4 levels, and the association with UC, the initial impression favored a diagnosis of type 2 AIP rather than type 1 AIP.

In contrast to adult AIP, there are no well-established guidelines for the diagnosis and treatment of pediatric AIP. However, recent guidelines suggest pediatric AIP should be diagnosed based on the combination of specific clinical symptoms (usually abdominal pain, jaundice, weight loss, fatigue, and vomiting in contrast to painless jaundice in adults) and cross-sectional imaging findings (focal pancreas enlargement, main pancreatic duct irregularities, and distal CBD narrowing). The diagnosis can be complimented by histopathological findings of acute and/or chronic inflammatory cell infiltration around the pancreatic acini and/or presence of IgG4-positive plasma cells with or without pancreas fibrosis (10, 11). Elevated serum IgG4 is strongly suggestive of adult AIP (elevated in 65% of adult AIP type 1 and 25% of adult AIP type 2 patients); however, this finding is of limited diagnostic value in children (elevated in 22% of the pediatric AIP) (10). In our case, pancreatic biopsy showed storiform fibrosis with inflammatory cellular stroma, IgG4-positive cells (up to 50 cells/HFP), and destruction of the lobulated architecture of the pancreatic parenchyma indicating AIP type 1 (IgG-related) in the absence of elevated serum IgG4 levels. This suggests distinct AIP patterns may be observed in pediatric cases that do not fit into either category (10).

Children presenting with a pancreatic mass and biliary obstruction are more likely to have AIP than neoplasms (12). Neoplastic pancreatic tumors are rare in pediatric patients, and pancreatic neoplasia is uncommon in pediatric cases of biliary obstruction (13). Cytologic analysis of samples collected by fine-needle aspiration cytology has poor diagnostic accuracy in pediatric AIP, while transduodenal, laparoscopic biopsy can be used to distinguish children with AIP from those with neoplasia (13). In the present case, type 1 AIP was diagnosed by histological examination of surgical specimens while EUS-guided FNA biopsy demonstrated atypical cells.

Corticosteroid therapy can be trialed prior to biopsy as an immediate response to corticosteroid therapy is characteristic of AIP (11). However, there was no response to corticosteroid therapy in the present case. This may have been due to massive necrosis and fibrosis that had already progressed to an advanced stage that was unlikely to respond to corticosteroid therapy. Treatment options are limited in pediatric IBD patients, and drug-induced pancreatitis should be carefully excluded to avoid unnecessary discontinuation of medications (14). In the present case, we initially discontinued mesalazine and AZA due to concerns regarding drug-induced pancreatitis. However, considering the subsequent diagnosis of type 1 AIP, discontinuation of these drugs may have been unnecessary.

The development of AIP is thought to be associated with the disease activity of IBD. It has been reported that recurrent episodes of acute pancreatitis are associated with higher disease activity scores in children with IBD, suggesting that pancreatic involvement may be associated with active disease (15). Furthermore, IBD has a more severe disease course when it is associated with AIP, and has a poor prognosis such as refractory colitis requiring colectomy (3). Meanwhile, in a recent study in adult patients with IBD, it has been suggested that there are two groups of UC patients who are at risk of AIP, one with rectal location and mild disease and the second with extensive and active disease not responding to biologic treatments (6). Regarding relapse, the recurrence rate of AIP varies from 6 to 55%, significantly higher in type 1 than 2 AIP (6, 16, 17). Considering that the development of AIP is associated with the disease activity of IBD, tight control of IBD progression is required to minimize the recurrence of AIP.

In conclusion, we provide the first report of a case of type 1 AIP in an adolescent with UC presenting with acute pancreatitis and a pancreatic mass mimicking pancreatic cancer. Pediatric gastroenterologists should keep in mind that type 1 AIP may present as a rare mass during the disease course of IBD. Moreover, tight control of IBD progression is required in order to minimize the occurrence of rare extra-intestinal manifestation of IBD such as AIP.

## Data Availability Statement

The original contributions presented in the study are included in the article/supplementary material, further inquiries can be directed to the corresponding author/s.

## Ethics Statement

The studies involving human participants were reviewed and approved by Institutional Review Board of Kyungpook National University Chilgok Hospital. Written informed consent to participate in this study was provided by the participants' legal guardian/next of kin.

## Author Contributions

SC and HL contributed in the acquisition, analysis and interpretation of data, and drafting of the initial manuscript. AS, HB, HK, CC, SL, and B-HC contributed in the acquisition, analysis and interpretation of data, and critical revision for important intellectual content. BK contributed in the conception of the case report, acquisition, analysis and interpretation of data, drafting of the initial manuscript, and critical revision for important intellectual content. All authors contributed to the article and approved the submitted version.

## Funding

This work was supported by the National Research Foundation of Korea (NRF) grant funded by the Korean government (MSIT) (No. 2021R1A2C1011004).

## Conflict of Interest

The authors declare that the research was conducted in the absence of any commercial or financial relationships that could be construed as a potential conflict of interest.

## Publisher's Note

All claims expressed in this article are solely those of the authors and do not necessarily represent those of their affiliated organizations, or those of the publisher, the editors and the reviewers. Any product that may be evaluated in this article, or claim that may be made by its manufacturer, is not guaranteed or endorsed by the publisher.

## Abbreviations

AIP: Autoimmune pancreatitis
CRP: C-reactive protein
CT: Computed tomography
FC: Fecal calprotectin
FIT: Fecal immunochemical testing
FNA: Fine needle aspiration
IBD: Inflammatory bowel disease
ICDC: International Consensus Diagnostic Criteria
UC: Ulcerative colitis.
